# Maternal feeding knowledge and practices as determinants of nutritional status in under-five children

**DOI:** 10.6026/973206300222020

**Published:** 2026-04-30

**Authors:** Jeevitha Solur Venkatesh, Meenal Kulkarni, K Senthil Kumar

**Affiliations:** 1 Department of Medicine, Sapthagiri Institute of Medical Science, Karnataka, India; 2 Department of Community Medicine, Professor, NKP Salve Institute of Medical Sciences & RC and Lata Mangeshkar Hospital, Nagpur, India; 3 Department of Physiology, Madha Medical College & RI, Tamil Nadu, India

**Keywords:** Maternal education, child malnutrition, knowledge, attitude and practice (KAP), dietary diversity, complementary feeding, anthropometric measurements, child growth, nutrition, maternal knowledge, nutritional practices, under-five children

## Abstract

Under-five malnutrition remains a persistent public health burden in low- and middle-income countries, contributing substantially to morbidity and impaired growth. Therefore, it is of interest to evaluate maternal knowledge, attitude and feeding practices (KAP) and examined their association with anthropometric indicators among children aged 6 months to 5 years. Maternal KAP was assessed using a structured questionnaire and child nutritional status was determined using weight-for-age, height-for-age and mid-upper arm circumference based on WHO standards. Moderate knowledge (53.3%) and practices (56.7%) were observed, while 23.3% were underweight, 20% stunted and 13.3% wasted. Thus, maternal feeding practices demonstrated significant positive correlations with weight-for-age (r=0.52), height-for-age (r=0.47) and MUAC (r=0.50), indicating behavioral determinants strongly influence child growth outcomes.

## Background:

Malnutrition remains a leading contributor to morbidity and mortality among children under five years, particularly in low- and middle- income countries [1]. Recent evidence indicates that underweight, stunting and wasting continue to affect a substantial proportion of children despite ongoing nutrition programs [2, 3]. Early childhood nutrition is crucial for optimal physical growth, neurocognitive development and immune competence [4]. Inadequate dietary diversity, delayed complementary feeding and improper feeding frequency are persistent determinants of under nutrition [5]. Maternal knowledge and caregiving practices significantly influence child feeding behaviors and household dietary decisions [6]. Studies have shown that insufficient maternal nutrition literacy is associated with poor growth indicators and higher prevalence of stunting and wasting [7, 8]. However, knowledge alone does not consistently translate into appropriate feeding practices, indicating behavioral and contextual barriers [9]. Attitudinal factors and maternal self-efficacy further modify adherence to recommended feeding guidelines [10]. Anthropometric indicators such as weight-for-age, height-for-age and mid-upper arm circumference remain standardized measures for evaluating nutritional status and growth outcomes [3, 4]. Examining the relationship between maternal KAP and objective anthropometric indices provides measurable insight into behavioral determinants of child nutrition [6, 8]. Despite expanded public health initiatives, gaps persist between awareness and actual implementation of optimal feeding practices [5, 9]. Therefore, it is of interest to evaluate maternal knowledge, attitude and feeding practices and analyse their association with anthropometric indicators among under-five children.

## Materials and Methods:

This cross-sectional descriptive study was conducted among mothers of children aged 6 months to 5 years attending selected community health centers and pediatric outpatient clinics. The sample size was estimated using prevalence data from recent maternal nutrition knowledge studies, targeting a minimum of 150 mother-child dyads to ensure adequate statistical power. Participants were recruited using purposive sampling based on eligibility criteria. Mothers with children within the specified age group who provided informed consent were included. Maternal knowledge, attitude and feeding practices were assessed using a pre-tested structured questionnaire. The knowledge component evaluated awareness of breastfeeding, complementary feeding, dietary diversity and meal frequency. The attitude section assessed perceptions toward recommended child feeding behaviors. The practice section captured actual feeding frequency, food variety, hygiene practices and complementary feeding initiation. The questionnaire was pilot tested among a subset of mothers to assess clarity and internal consistency. Necessary modifications were made prior to final data collection. Child anthropometric measurements were obtained following WHO standardized protocols. Weight was measured using a calibrated digital weighing scale with minimal clothing. Length or height was measured using an infantometer or stadiometer as appropriate for age. Mid-upper arm circumference (MUAC) was measured using a non-stretchable measuring tape. Weight-for-age, height-for-age and weight-for-height Z-scores were calculated using WHO growth standards. Underweight, stunting and wasting were defined as Z-scores below -2 standard deviations. Data were entered and analysed using statistical software. Descriptive statistics summarized maternal KAP levels and anthropometric categories. Pearson correlation analysis assessed the association between maternal practice scores and anthropometric indicators. Statistical significance was set at p < 0.05. Ethical approval was obtained from the institutional ethics committee. Written informed consent was secured from all participants. Confidentiality and anonymity were strictly maintained throughout the study.

## Results:

A total of 150 mother-child dyads were included in the analysis. The largest proportion of children was aged 13-24 months (26.7%). Most mothers had secondary-level education (36.7%). Moderate knowledge (53.3%), neutral attitudes (46.7%) and moderate feeding practices (56.7%) were predominant. Underweight, stunting and wasting were observed in 23.3%, 20.0% and 13.3% of children, respectively. Significant positive correlations were identified between maternal practices and anthropometric indicators. Children of mothers with higher KAP scores demonstrated better nutritional status. Table 1 (see PDF) shows that the highest proportion of children (26.7%) was aged 13-24 months, while the lowest proportion (16.7%) were aged 6-12 months. Table 2 (see PDF) indicates that 36.7% of mothers had secondary education, whereas 10% had no formal education. Table 3 (see PDF) demonstrates that 53.3% of mothers had average knowledge scores, while 13.3% had poor knowledge. Table 4 (see PDF) highlights that 46.7% of mothers had neutral attitudes, compared to 16.7% with negative attitudes. Table 5 (see PDF) shows that 56.7% of mothers exhibited moderate feeding practices, while 20% demonstrated poor practices. Table 6 (see PDF) indicates that 23.3% of children were underweight, whereas 66.7% had normal weight-for-age. Table 7 (see PDF) demonstrates that 20% of children were stunted, while 76.7% had normal height-for-age. Table 8 (see PDF) shows that 13.3% of children were wasted compared to 76.7% with normal weight-for-height. Table 9 (see PDF) indicates significant positive correlations between maternal practices and weight-for-age (r=0.52), height-for-age (r=0.47) and MUAC (r=0.50), all with p<0.01. Table 10 (see PDF) demonstrates that undernutrition was more frequent among children of mothers with poor KAP, while normal nutrition was predominant among those with good KAP.

## Discussion:

This study examined maternal knowledge, attitude and feeding practices and their association with anthropometric outcomes among under- five children [11]. Moderate knowledge and practice levels were predominant, while a substantial proportion of children remained underweight, stunted, or wasted [12]. Similar findings have been reported in recent community-based studies showing persistent undernutrition despite moderate maternal awareness [13]. These findings suggest that knowledge alone may not be sufficient to ensure optimal nutritional outcomes. Although over half of mothers demonstrated average knowledge, gaps were observed in complementary feeding and dietary diversity practices. Recent evidence indicates that inadequate dietary diversity and delayed complementary feeding remain strong predictors of stunting and wasting [14]. Studies have shown that maternal nutrition literacy improves feeding quality only when accompanied by behavioral reinforcement and social support [15]. This may explain the moderate translation of knowledge into practice observed in the present study. Maternal attitudes were largely neutral, which may reflect limited self-efficacy or socio-cultural constraints affecting feeding decisions. Contemporary research highlights that positive maternal attitudes and confidence significantly influence adherence to recommended feeding guidelines [16]. Behavioral determinants such as household food security, time constraints and cultural norms further mediate feeding practices [17]. Therefore, attitude and contextual factors remain critical in shaping child nutrition outcomes. The prevalence of underweight (23.3%), stunting (20%) and wasting (13.3%) in this study is consistent with recent regional reports from similar settings [18]. Despite ongoing nutrition programs, undernutrition continues to pose a significant public health challenge [19]. These findings reinforce the need for targeted behavioral interventions rather than knowledge-based education alone. A key finding was the significant positive correlation between maternal practices and anthropometric indicators, including weight-for-age, height-for-age and MUAC. Comparable studies have demonstrated that improved feeding practices are directly associated with better growth indices [20]. The strength of correlation observed in this study underscores the measurable impact of maternal behavior on child growth outcomes. This provides objective anthropometric evidence linking caregiving practices to nutritional status. Importantly, children of mothers with poor KAP scores were more likely to be undernourished, whereas those with good KAP scores were predominantly normally nourished. Recent longitudinal analyses have similarly identified maternal practice as an independent predictor of nutritional status [21]. By integrating subjective KAP assessment with objective anthropometric measures, this study strengthens the behavioral-biological linkage in child nutrition research. This contributes contemporary evidence emphasizing that practice-oriented interventions may yield measurable improvements in growth indicators. Community-based nutrition strategies should therefore prioritize behavior modification, complementary feeding counseling and growth monitoring. Interventions combining maternal education with practical skill-building have demonstrated improved dietary diversity and reduced stunting rates [22]. Strengthening maternal empowerment and consistent anthropometric surveillance may enhance early detection and prevention of under nutrition. Overall, this study reinforces that maternal feeding practices are significant determinants of child anthropometric outcomes. Bridging the gap between knowledge and behavior remain essential for sustainable improvement in under-five nutritional status.

## Conclusion:

Under-five malnutrition persists despite moderate maternal knowledge and attitudes, indicating that awareness alone are insufficient to improve child growth outcomes. Maternal feeding practices showed significant positive associations with anthropometric indicators, providing objective evidence that behavior directly influences nutritional status. Thus, targeted, practice-oriented nutrition interventions and strengthened maternal empowerment are imperative to translate knowledge into measurable improvements in child growth and development.
